# Neural stem cells express melatonin receptors and neurotrophic factors: colocalization of the MT_1 _receptor with neuronal and glial markers

**DOI:** 10.1186/1471-2202-5-41

**Published:** 2004-10-28

**Authors:** Lennard P Niles, Kristen J Armstrong, Lyda M Rincón Castro, Chung V Dao, Rohita Sharma, Catherine R McMillan, Laurie C Doering, David L Kirkham

**Affiliations:** 1 Department of Psychiatry and Behavioural Neurosciences, McMaster University 1200 Main Street West, Hamilton ON L8N 3Z5, Canada; 2 Department of Pathology and Molecular Medicine, McMaster University 1200 Main Street West, Hamilton ON L8N 3Z5, Canada

## Abstract

**Background:**

In order to optimize the potential benefits of neural stem cell (NSC) transplantation for the treatment of neurodegenerative disorders, it is necessary to understand their biological characteristics. Although neurotrophin transduction strategies are promising, alternative approaches such as the modulation of intrinsic neurotrophin expression by NSCs, could also be beneficial. Therefore, utilizing the C17.2 neural stem cell line, we have examined the expression of selected neurotrophic factors under different *in vitro *conditions. In view of recent evidence suggesting a role for the pineal hormone melatonin in vertebrate development, it was also of interest to determine whether its G protein-coupled MT_1 _and MT_2 _receptors are expressed in NSCs.

**Results:**

RT-PCR analysis revealed robust expression of glial cell-line derived neurotrophic factor (GDNF), brain-derived neurotrophic factor (BDNF) and nerve growth factor (NGF) in undifferentiated cells maintained for two days in culture. After one week, differentiating cells continued to exhibit high expression of BDNF and NGF, but GDNF expression was lower or absent, depending on the culture conditions utilized. Melatonin MT_1 _receptor mRNA was detected in NSCs maintained for two days in culture, but the MT_2 _receptor was not seen. An immature MT_1 _receptor of about 30 kDa was detected by western blotting in NSCs cultured for two days, whereas a mature receptor of about 40 – 45 kDa was present in cells maintained for longer periods. Immunocytochemical studies demonstrated that the MT_1 _receptor is expressed in both neural (β-tubulin III positive) and glial (GFAP positive) progenitor cells. An examination of the effects of melatonin on neurotrophin expression revealed that low physiological concentrations of this hormone caused a significant induction of GDNF mRNA expression in NSCs following treatment for 24 hours.

**Conclusions:**

The phenotypic characteristics of C17.2 cells suggest that they are a heterogeneous population of NSCs including both neural and glial progenitors, as observed under the cell culture conditions used in this study. These NSCs have an intrinsic ability to express neurotrophic factors, with an apparent suppression of GDNF expression after several days in culture. The detection of melatonin receptors in neural stem/progenitor cells suggests involvement of this pleiotropic hormone in mammalian neurodevelopment. Moreover, the ability of melatonin to induce GDNF expression in C17.2 cells supports a functional role for the MT_1 _receptor expressed in these NSCs. In view of the potency of GDNF in promoting the survival of dopaminergic neurons, these novel findings have implications for the utilization of melatonin in neuroprotective strategies, especially in Parkinson's disease.

## Background

Neural stem cells are multipotent cells which are capable of self-replication and differentiation into neurons, astrocytes or oligodendrocytes in the central nervous system [1]. Because of their intrinsic plasticity and multipotency, there are great expectations that NSC transplantation will ultimately provide immense benefits in the treatment of neurodegeneration. However, it is essential to fully understand the cellular and molecular mechanisms involved in the differentiation and function of NSCs, in order to fully harness their therapeutic potential. Because of the very limited availability of NSCs in the central nervous system (CNS), neural stem cell lines are very useful for the study and characterization of NSC biology. For example, transplantation studies with the C17.2 neural stem cell line [2] have revealed that these cells express diverse neurotransmitter phenotypes, depending on the environment prevailing in the CNS area of engraftment [3,4]. Recently, transplanted C17.2 NSCs, genetically modified to express glial cell line-derived neurotrophic factor (GDNF), were found to engraft in the 6-hydroxydopamine-lesioned mouse striatum and to express therapeutic levels of this neurotrophin, with consequent protection of dopaminergic neurons in this model of Parkinson's disease [5]. Although this and other similar approaches are promising, limitations including the stability and regulation of transduced genes await resolution. Therefore, it was of interest to determine whether C17.2 cells have the intrinsic ability to express neurotrophins or neurotrophic factors, which would make them amenable to modulation by appropriate agents *in vitro or in vivo*. In addition, we examined whether these NSCs express receptors for the pineal hormone melatonin, which can induce GDNF mRNA and protein expression [6,7] and which has been implicated in the development of vertebrates including humans [8-10]. Initially, different concentrations and types of sera were used for cell culture in order to select optimal conditions for gene expression studies. We now report that C17.2 NSCs exhibit heterogeneous phenotypes and express neurotrophic factors and melatonin MT_1 _receptors.

## Results

### Effects of culture conditions on neurotrophic factor and cell-specific marker mRNA expression in C17.2 NSCs

**Figure 1:**
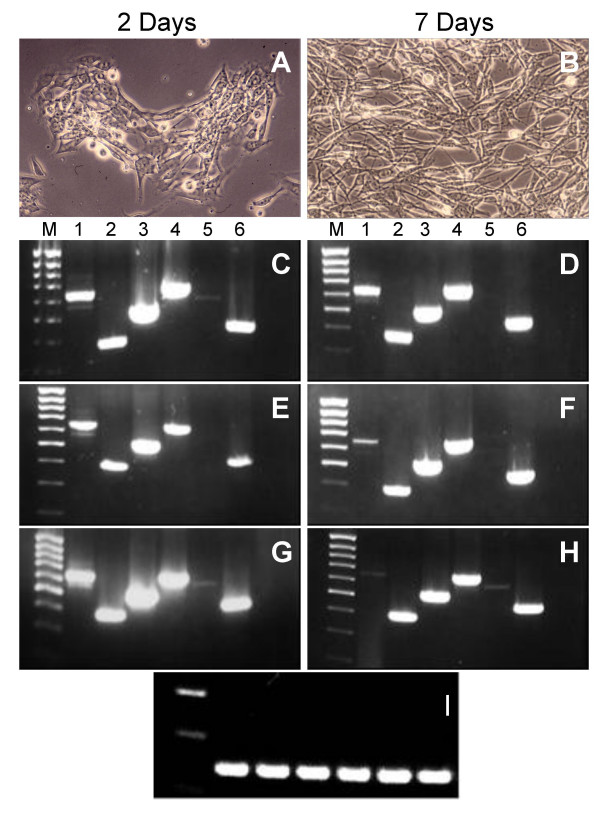
**Morphology and expression of neurotrophins and phenotypic markers in C17.2 NSCs. **(A) Undifferentiated cells exhibit a flat and rounded structure after 2 days in culture, as revealed by phase contrast microscopy. (B) After 7 days in culture, differentiating cells appear elongated with processes. Lanes:1–6: GDNF, BDNF, NGF, Nestin, GFAP and β-tubulin III mRNA expression in neural stem cells maintained in 1% calf serum (C,D), 1% fetal bovine serum (E,F), or 10% fetal bovine serum + 5% horse serum (G,H) for 2 or 7 days as indicated. (I) GAPDH mRNA from cells cultured in 1% calf serum, 1% fetal bovine serum (FBS) or 10% FBS + 5% horse serum (HS)-Lanes: 1–3 (2 days), 4–6 (7 days).

Following two days in culture, C17.2 cells remain in an undifferentiated state, as indicated by their flat and rounded appearance (Fig. 1A) and high expression of the stem cell/progenitor cell marker, nestin (Fig. 1C,1E,1G). These cells also expressed the early neuronal marker, β-tubulin III, but there was little or no expression of the mRNA for the glial marker, glial fibrillary acidic protein (GFAP). After seven days in culture, differentiating C17.2 cells exhibit an elongated shape with an extension of neurite-like processes, as shown in Fig. 1B. However, as observed in undifferentiated cells after two days, there was still strong expression of nestin and β-tubulin III, with little or no detectable GFAP mRNA (Fig. 1D,1F,1H). An examination of neurotrophin mRNA expression in undifferentiated C17.2 cells, revealed a robust expression of GDNF, BDNF and NGF, regardless of the type or concentration of serum used for culturing (Fig. 1C,1E,1G). A similar strong expression of BDNF and NGF was observed in differentiating cells after seven days, but GDNF mRNA was relatively lower in cells maintained in 1% fetal bovine serum (FBS) or 10% FBS + 5% horse serum (Fig. 1F,1H). The mRNA levels of the control gene, GAPDH, did not change under the conditions examined (Fig. 1I).

### Detection of MT_1 _receptor mRNA and protein in C17.2 NSCs

**Figure 2:**
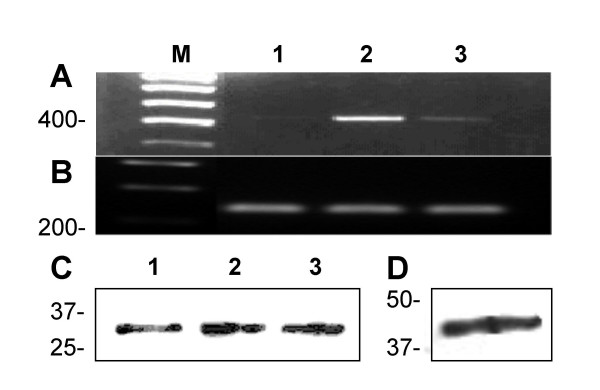
**Melatonin MT_1 _receptor mRNA and protein expression in C17.2 NSCs. **(A) RT-PCR detection of the MT_1 _transcript (397 bp) in neural stem cells (NSCs) maintained for 2 days in 1% FBS: lane 2 or 10% FBS + 5%HS: lane 3, but not in 1% calf serum : lane 1. (B) Expression of glyceraldehyde- 3-phosphate dehydrogenase (GAPDH, 237 bp), lanes 1–3. (C) Cells were kept in culture for the number of days indicated and extracted proteins were examined by western analysis. Lane 1: 1 day in 1% FBS; lane 2: 1 day in 10% FBS + 5% HS; lane 3: 3 days in 1% FBS. (D) Cells were grown for 1 week in 10% FBS + 5% HS and then subcultured in 1% FBS for 5 days before western analysis. Molecular weight (kDa) markers are indicated on the left.

Melatonin MT_1 _receptor mRNA was detected by RT-PCR in NSCs maintained for two days, especially in cells cultured in 1% FBS, as shown in Fig. 2A. GAPDH mRNA expression did not change under the conditions examined (Fig. 2B). C17.2 NSCs maintained for indicated periods in 1% FBS or 10% FBS + 5% horse serum, expressed the MT_1 _receptor protein, as revealed by western analysis (Fig. 2C,2D). Interestingly, when cells were cultured for 2–3 days, the MT_1 _protein detected had a molecular weight of about 30 kDa, which is less than the predicted size of the mature receptor. However, when cells were cultured for 10–12 days, a mature MT_1 _receptor of about 40–45 kDa was present, as shown in Fig. 2D. The MT_2 _receptor transcript was not detected under any of the conditions used in this study.

### Immunocytochemical detection of the MT_1 _receptor and cell-specific markers in C17.2 NSCs

**Figure 3:**
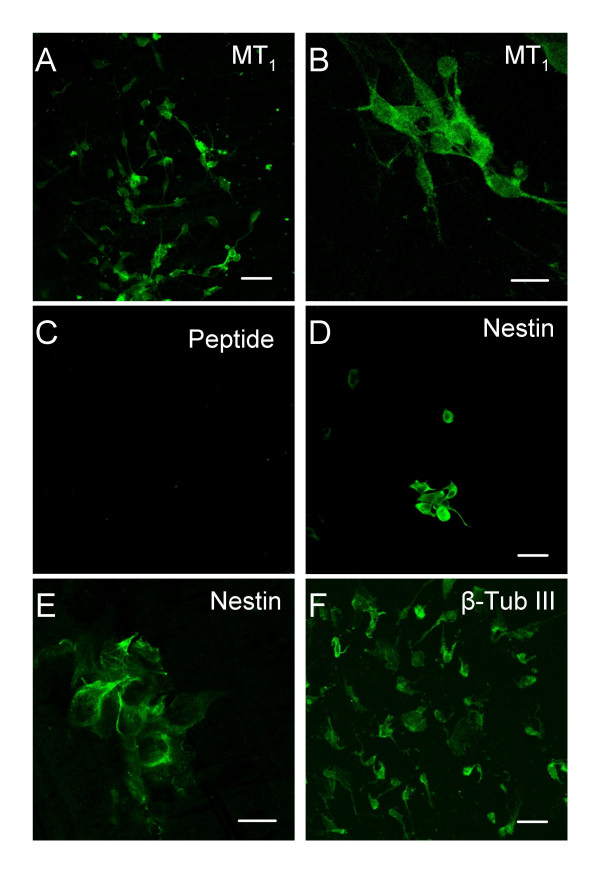
**Melatonin MT_1 _receptor, nestin and β-tubulin III immunoreactivity in C17.2 NSCs. **Cells were maintained in 1% FBS for the number of days indicated, except for those shown in panel E, which were cultured in 10% FBS + 5% HS. (A,B) MT_1 _in cells maintained for 2 days. (C) Peptide (immunogen) blockade of MT_1 _immunoreactivity after 2 days. (D,E) Nestin immunoreactivity after 2 and 3 days, respectively. (F) β-tubulin III immunoreactivity after 3 days. Scale bars: (A,D,F) 50 μm. (B,E) 20 μm.

**Figure 4:**
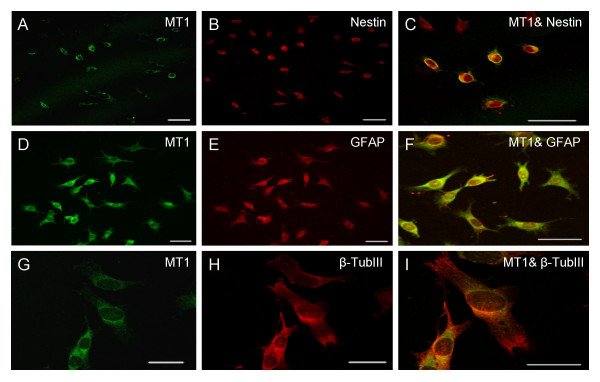
**Colocalization of the MT_1 _receptor with phenotypic markers in C17.2 NSCs. **Cells were maintained in 10% FBS + 5% HS for 9 days and then in 1% FBS for 3 days before seeding on coverslips in 1% FBS for 2 days. (A,B,C) Confocal images of MT_1 _(green), Nestin (red), and MT_1 _& nestin. (D,E,F) MT_1 _(green), GFAP (red) and MT_1 _& GFAP. (G,H,I) MT_1 _(green), β-tubulin III (red) and MT_1 _& β-tubulin III. Double-labeled cells exhibit yellow-orange fluorescence. Scale bars: A-F: 50 μm; G-I: 20 μm.

MT_1 _receptor immunoreactivity was detected within C17.2 cells maintained in 1% FBS for two days, as shown in Figure 3A,3B. Omission of the primary antibody or its preincubation with a blocking peptide (CIDtech Research Inc., Cambridge, ON) abolished MT_1 _immunoreactivity (Fig. 3C), indicating the specificity of MT_1 _detection. In keeping with RT-PCR results, nestin (Fig. 3D,3E) and β-tubulin III (Fig. 3F), were detected by immunocytochemical analysis. Double- labeling studies indicated that the MT_1 _receptor is coexpressed with the stem /progenitor cell marker, nestin (Fig. 4A,4B,4C), the glial marker, GFAP (Fig. 4D,4E,4F) and the early neuronal marker, β-tubulin III (Fig. 4G,4H,4I).

### Induction of GDNF mRNA expression by melatonin in C17.2 NSCs

**Figure 5:**
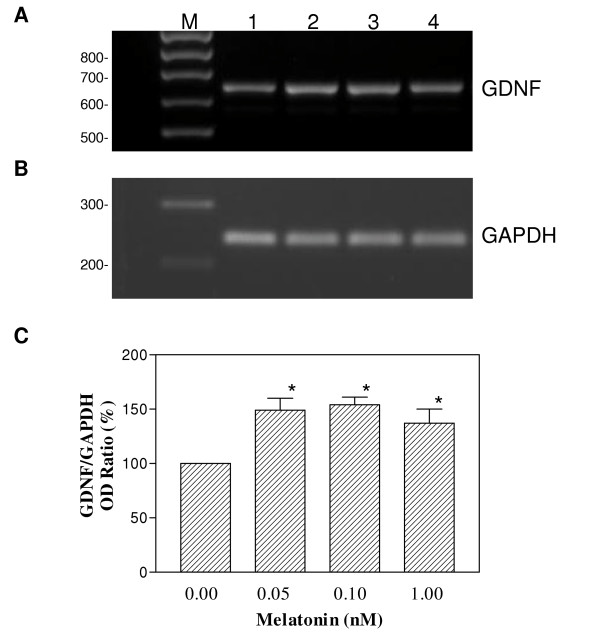
**Induction of GDNF mRNA by melatonin in C17.2 NSCs. **(A,B) Gel images of RT-PCR amplification of GDNF (643 bp) and GAPDH (237 bp). Lanes 1–4: Control, 0.05, 0.1, and 1 nM melatonin. M = markers. (C) Percentage values of GDNF/GAPDH optical density ratios as a function of melatonin treatment. Data shown are the means ± s.e.m. from 2 separate experiments. *p < 0.05 vs control.

In order to assess the potential functionality of the MT_1 _receptor detected in C17.2 NSCs, the effect of low physiological concentrations of melatonin on GDNF mRNA expression was examined. Cells were grown as described in Methods and treated with melatonin or vehicle (0.001% DMSO) for 24 hours. Following RT-PCR analysis, GDNF mRNA levels were converted to optical density (OD) values and normalized to GAPDH OD levels, as reported previously [6]. After conversion of GDNF/GAPDH OD ratios to percentage values, one-way ANOVA indicated a significant treatment effect (F_3,7 _= 7.03, p < 0.04). A Neuman-Keuls test indicated a significant increase in relative GDNF mRNA expression in cells treated with 0.05, 0.1 and 1 nM melatonin as shown in Figure 5.

## Discussion

The expression of nestin in undifferentiated C17.2 cells is consistent with the presence of this intermediate filament protein in stem and progenitor cells in the mammalian CNS [11]. However, as noted above, nestin mRNA was also readily detected in cells exhibiting morphological changes characteristic of differentiation, after one week in culture. Similarly, mRNA for the early neuronal marker, β-tubulin III, was found under all conditions examined, whereas GFAP mRNA was detected only in some cultures. These observations suggest that the C17.2 cells examined in this study are an heterogeneous population of stem and progenitor cells in keeping with evidence that NSCs exhibit morphological and phenotypic heterogeneity [12,13].

The expression of diverse neurotrophins by NSCs is consistent with the role of these factors in the differentiation and development of the CNS. Presumably, the robust mRNA expression observed, particularly in cells maintained in 10% FBS + 5% HS, is driven by the serum-enriched milieu of potential inducers including neurotransmitters, hormones and growth factors, such as basic fibroblast growth factor and epidermal growth factor, which can stimulate C17.2 cell growth *in vitro *[14]. In contrast to BDNF and NGF, which exhibited strong mRNA expression under all conditions examined, GDNF expression was weaker or not detectable in differentiating cells after seven days in culture. The suppression of GDNF expression might have been due to the prolonged exposure of NSCs to regulatory factors in the serum, as its decline appears to be inversely correlated with the concentration or enrichment of serum used for cell culture. Thus, moderate, weak or no expression of GDNF was observed in cells cultured for 1 week in 1% CS, 1% FBS or 10% FBS + 5% HS, respectively (see Fig. 1D,1F,1H). Various biological agents or pathways have been implicated in the regulation of GDNF expression. For example, fibroblast growth factor-2 and proinflammatory cytokines such as interleukin(IL)-1β, IL-6 and tumor necrosis factor-α stimulate GDNF synthesis and secretion [15]. Activation of protein kinase C by phorbol esters increases GDNF expression [15,16], whereas the adenylate cyclase activator, forskolin, inhibits GDNF production in cultured cells, suggesting an inhibitory role for the cyclic AMP- protein kinase A pathway [15]. The cAMP pathway and its transcriptional factor cAMP response element binding protein (CREB) have been shown to induce differentiation in neuronal progenitor cells [17,18]. Therefore, it is possible that activation of this pathway was involved in both the initiation of differentiation and the inhibition of GDNF expression observed in C17.2 cells after seven days.

While this work was in progress, it was reported that C17.2 neural stem cells constitutively secrete BDNF, GDNF and NGF, but do not label for GFAP or neuronal markers like β-tubulin III [19]. Our findings are in agreement with these observations with regard to neurotrophin expression. However, in contrast to their findings, β-tubulin III mRNA and immunoreactivity were readily detected in our study. In addition, although GFAP mRNA was weakly expressed or not detectable in some cultures, immunoreactivity for this glial cell marker is present in C17.2 cells, as shown in Figure 4. These differences may be due to our examination of β-tubulin III and GFAP expression in cells maintained for 2–12 days in culture, whereas their C17.2 cells were examined after 2–3 weeks [19]. Other factors, such as our use of low serum concentrations, as compared with the enriched culture medium used by Lu et al. [19], may also be involved. The detection of melatonin MT_1 _receptor mRNA in C17.2 cells after 2 days but not after 7 days, presumably involves downregulation of this receptor. There is considerable evidence that many G protein-coupled receptors are downregulated by their agonists [20]. More importantly, melatonin, which is present in serum, has been found to suppress MT_1 _transcription *in vitro *[21]. Interestingly, our immunocytochemical studies revealed MT_1 _immunoreactivity within C17.2 cell bodies and extensions, as shown in Figure 3A,3B. Although an intracellular localization could result from internalization of receptors [20], it is also possible that the immunoreactivity detected within these neural stem/progenitor cells is due to the presence of newly synthesized MT_1 _receptors. In accordance with this view, the MT_1_ protein detected in short-term (2-day) cultures is about 30 kDa, which is less than the approximately 37–45 kDa molecular weight observed in various mammalian tissues [22-24]. Moreover, when cells were cultured for 10–12 days, a MT_1 _receptor of about 40–45 kDa was detected, as shown in Fig. 2D. The mammalian MT_1 _contains two glycosylation sites in its N-terminal [24] and it may exist in more than one glycosylated form, as has been reported for other G protein-coupled receptors [25,26]. Thus, the above cytochemical observations suggest that newly synthesized immature MT_1 _receptors,which have yet to undergo posttranslational modification and translocation to the plasma membrane, were detected in cells cultured for 2 days in 1% FBS, whereas a mature glycosylated receptor was present in cells grown for longer periods. Although the MT_2 _receptor transcript was not detected under any of the conditions used in this study, additional studies are required before the possibility of its expression in these cells can be ruled out. It is possible that MT_2 _mRNA may undergo rapid turnover/degradation, while a functional protein may still be present. This is the first evidence that melatonin receptors are expressed in neural stem or progenitor cells and raises the obvious question of whether this hormone plays a role in neuronal development. Although studies in this field are limited, there is increasing evidence that melatonin is involved in the early development of vertebrates. For example, melatonin is produced in chick embryos as early as the 7^th ^day of embryonic development [27], and a physiological concentration of this hormone has been shown to significantly enhance mouse embryogenesis *in vitro *[8]. Similarly, when sheep blastocysts were treated with melatonin for 24 hr *in vitro*, there was a significant increase in the percentage of embryonic survival [28]. Other studies have shown that functional G_i _protein-coupled melatonin receptors, which mediate inhibition of the adenylate-cyclase-cAMP pathway, are present in the embryonic (day 8) neural retina [29]. Melatonin receptor transcripts for all the known G_i _protein-coupled receptor subtypes have been found in 24 hr-old embryos from Japanese quail [30]. Various studies have detected melatonin receptors in human fetal brain [31,32] and peripheral tissues [33]. Moreover, recent autoradiographic and *in situ *hybridization studies indicate that the melatonin MT_1 _receptor is expressed in diverse areas of the human fetal brain [9]. Thus, the presence of MT_1 _receptors in NSCs is in keeping with the foregoing, and supports the view that melatonin is involved in neurodevelopment. Colocalization evidence that the MT_1 _receptor is present in both neural and glial progenitor cells is consistent with a neurodevelopmental role for melatonin, and suggests that in addition to the presence of the MT_1 _in mammalian neurons [34], it may also be expressed in astrocytes, as observed in similar cells from rat [6] and chick brain [35]. The detection of nestin in some cells expressing the MT_1 _receptor is consistent with its presence not only in neural progenitor cells but also in GFAP positive glial progenitors [36]. Preliminary evidence that melatonin induces GDNF mRNA expression in C17.2 NSCs, as we have observed previously in C6 glioma cells [6], supports the foregoing as this neurotrophic factor plays a critical role in both central and peripheral neurodevelopment [37,38]. GDNF also exerts neuroprotective effects in the CNS, including a potent role in the survival of dopaminergic neurons in the midbrain [39,40]. Therefore, modulation of GDNF expression may be one of the mechanisms underlying physiological neuroprotection by melatonin in the CNS [6].

## Conclusions

In summary, the NSCs utilized in this study exhibited an intrinsic ability to express neurotrophins under various cell culture conditions. This ability was not affected by their morphological state, except in the case of GDNF mRNA expression which was lower in cells undergoing differentiation in FBS-supplemented media. Novel evidence that neural stem/progenitor cells express MT_1 _receptors adds to the increasing evidence that NSCs can respond to diverse modulators [41], and suggests an early role for melatonin in CNS development. Moreover, since melatonin induces GDNF expression in NSCs, its potential *in vivo *modulation of this and/or other neurotrophic factors, via its G protein-coupled receptors in the brain or on transplanted NSCs, could have important implications for optimizing therapeutic strategies in neurodegenerative disorders such as Parkinson's disease.

## Methods

### Cell culture

The C17.2 cell line was derived by retrovirus-mediated oncogene (*v-myc*) transduction of cells from the external germinal layer of neonatal mouse cerebellum [2]. C17.2 cells were grown on 10 cm Corning culture dishes (Fisher Scientific Ltd., Nepean, ON, Canada) in DMEM supplemented with 2 mM glutamine and calf serum, fetal bovine serum or horse serum (Invitrogen Canada Inc., Burlington, ON) in the concentrations indicated. Cells were maintained in a humidified 5% CO_2 _– 95% air incubator at 37°C and routinely split at approximately 90% confluency [3].

### RT-PCR

Total RNA was isolated from C17.2 cells with TRIzol as described by the supplier (Invitrogen Canada Inc., Burlington, ON). After DNase treatment, cDNA was synthesized from 1–2 μg of total RNA using the Omniscript reverse transcriptase kit (Qiagen Inc., Mississauga, ON) and oligo dT primers. PCR was carried out using 1.5 μl (or 3 μl for melatonin MT_1 _and MT_2 _receptors) of the RT product and the HotStarTaq master mix kit (Qiagen Inc., Mississauga, ON), together with appropriate primers (Table 1). Following a hot start at 95°C for 15 min, samples were amplified for 36 cycles (or 38 cycles for MT_1 _and MT_2_) at 94°C for 30 s, 57°C for 30 s and 72°C for 1 min, followed by a final incubation at 72°C for 10 min.

### Treatment of C17.2 NSCs with melatonin

For semi-quantitative examination of the effects of melatonin on GDNF mRNA expression, cells were grown in 10% FBS + 5% horse serum (HS) for 1 week. After subculture, cells were kept in 10% FBS + 5% HS for 2 days followed by another subculture to 1% FBS for 2 or 3 days. The cells were then treated with vehicle (0.001% DMSO) or melatonin (0.05, 0.1, and 1 nM) for 24 hours. Following RNA extraction, RT-PCR was performed as described above, except that an annealing T_m _of 55°C was used and samples were amplified for 30 cycles. Glyceraldehyde 3-phosphate dehydrogenase (GAPDH), was amplified with intron-spanning primers [42], in order to control for DNA contamination.

### Immunocytochemistry

After fixation for 15 minutes in 4% paraformaldehyde on poly L-ornithine-coated glass cover slips, cells were incubated overnight at 4°C with anti-melatonin MT_1 _receptor serum (1:100; CIDtech Research Inc., Cambridge, ON). Cells were washed three times with PBS and then incubated with a fluorescein (FITC)-conjugated donkey anti- rabbit IgG (1:100 dilution; Jackson ImmunoResearch Labs. Inc.,West Grove, PA). In some experiments, the primary antibody was omitted or it was preincubated with the corresponding peptide immunogen (CIDtech Research Inc., Cambridge, ON), before use. In order to examine cell marker expression, mouse monoclonal antibodies against nestin (1: 500), β-tubulin III (1:200) or GFAP (1:400; Chemicon International, Temecula, CA) were used together with a FITC-conjugated donkey anti-mouse IgG (1:100; Jackson ImmunoResearch Labs.Inc.,West Grove, PA). For double- labeling studies of the MT_1 _and cell markers, a rhodamine (TRITC)-conjugated donkey anti-mouse IgG (1: 100; Jackson ImmunoResearch Labs. Inc.,West Grove, PA) was used to detect nestin, GFAP and β-tubulin III. Digital images were recorded on a Zeiss confocal microscope.

### Western analysis

C17.2 NSCs were grown as described in Figure 2, and proteins were extracted in a modified RIPA buffer (50 mM Tris-HCl pH 7.4, 150 mM NaCl, 1 mM EDTA, 1% NP-40, 0.25% sodium deoxycholate) supplemented with PMSF (1 mM), aprotinin (2 μg/ml), leupeptin (2 μg/ml), and sodium orthovanadate (2 mM). Extracted proteins (80 μg per lane) were separated by SDS- polyacrylamide gel electrophoresis and transblotted to nitrocellulose membranes. The blots were blocked with 5% nonfat dry milk in TBS-T buffer (50 mM Tris-HCl, 150 mM NaCl, 0.1% Tween 20; pH 8.5) for 1 hour at room temperature, and then incubated overnight with a 1:100 dilution of rabbit anti-MT_1 _antibody (CIDTech Research Inc., Cambridge, ON) at 4°C. After washing, membranes were incubated with a horseradish peroxidase-conjugated second antibody (1:1000; Santa Cruz Biotechnology, Inc., Santa Cruz, CA) for 1 hour. Following washing and exposure to enhanced chemiluminescence (ECL) reagents (Amersham Biosciences, Inc., Baie d'Urfé, Québec) for about 5 minutes, proteins were detected by autoradiography, as described previously [43]. Buffer reagents and protease inhibitors were obtained from Sigma- Aldrich Canada Ltd. (Oakville, ON).

## Authors' contributions

LPN conceived and planned the study, designed PCR primers, supervised all aspects of the study and wrote the manuscript. KJA cultured cells and performed initial RT-PCR experiments. LMRC treated cultured cells and carried out immunocytochemistry experiments. CVD cultured cells and carried out western blotting. RS treated cultured cells and performed RT-PCR experiments. CRM performed double- labeling of cultured cells. LCD provided the C17.2 cells, and collaborated on immunocytochemical studies. DLK assisted with initial cell culture. All authors read and approved the final manuscript.

## Figures and Tables

**Table 1:** Forward and reverse primers used for PCR amplification

**Gene**	**Primers (5'→3')**	**Nucleotides**	**Size(bp)**
GDNF	atgggatgtcgtggctgtctg	58–98	643
	tctctggagccagggtcagat	700–680	
BDNF	ggatgaggaccagaaggttgc	2342–2362	390
	ttgtctatgcccctgcagcct	2731-2711	
NGF	gcagacccgcaacatcactgt	484–504	517
	agccttcctgctgagcacaca	1000-980	
Nestin	aggaaccaaaagagacaggtg	4141–4161	653
	ttcctcagatgagaggtcaga	4793-4773	
GFAP	cctcaagaggaacatcgtggt	1119–1139	592
	acactggagtcatcacctgga	1710-1690	
β-Tubulin III	tagtggagaacacagacgaga	600–620	442
	ctgctgttcttactctggatg	1041-1021	
MT_1_	tgagtgtcatcggctcgatat	1–21	397
	tagtaactagccacgaacagc	397-377	
MT_2_	tgctgcatctgtcatagtacc	4–24	297
	acatggttaggaaactgcgca	346-326	
GAPDH	ttcaccaccatggagaaggc	1147–1166	237
	ggcatggactgtggtcatga	1383-1364	
